# Surgery

**Published:** 1892-12

**Authors:** James Swain


### SURGERY.

Operative procedures, in cases of enlarged prostate where catheter treatment has failed, or is unavailable, have been steadily gaining ground since the excellent address of McGill, at the meeting of the British Medical Association at Leeds, in 1889.[5] The most recent statistics of Bottini's treatment [6] by the galvano-cautery seem to show that 52 cases out of 77 were cured, 2 died, in 12 there was no result, and in 11 slight improvement. In these cases, however, the condition of the bladder before and after treatment has not been given; it is merely stated that the amount of residual urine was greatly reduced—a result which is not unusual under much simpler treatment. The operation appears useless in advanced disease, or for vesical growths (the most distressing form), or for enlargement of the lateral lobes. The balance of opinion seems decidedly in favour of either supra-pubic or perineal prostatectomy. McGill,[7] and Belfield [8] who collected 133 cases, did a great deal towards establishing prostatectomy as a recognised surgical procedure; but the recent lectures of Mansell Moullin, at the Royal College of Surgeons of England,[9] have placed the operation on a more definite basis than was possible with the earlier operators.

Perineal prostatectomy is certainly not so serious an operation as the supra-pubic operation. Of the former

[5] *Brit. Med. Journ.*, 1889, vol. ii., p. 863.

[6] An account of this operation will be found in the *Edinburgh Medical Journal*, March, 1891, p. 830.

[7] *Op. cit.* [8] *Amer. Journ. Med. Sc.*, Nov., 1890.

[9] *Brit. Med. Journ.*, 1892, vol. i., pp. 1185, 1250, 1294.

Moullin quotes 38 cases, with 3 deaths, *i.e.* 8 per cent., but one of these was 80 years of age already, and in another the method adopted was unsuitable; and of the supra-pubic operation, taking all the cases together, the mortality is approximately 20 per cent.; if the last half only, it is still 15 per cent. On the other hand, it must be remembered that the majority of patients operated upon by the latter method were practically *in extremis*, and that it is very doubtful whether a very large proportion would have survived much longer, or even so long, had the operation not been done.

Belfield's statistics [1] give the mortality of the perineal operation as 9 per cent., and of the supra-pubic as 16 per cent.

Moullin thinks the perineal operation to be chiefly of use in cases where it is possible to diagnose the presence of a small median outgrowth, the rest of the prostate being normal. "There has been sudden retention; a catheter passes in easily, showing that there is no congestion, but not a drop will come out. On examination by the rectum, the gland does not appear much enlarged, but there has been frequent micturition, with dribbling afterwards for some time. In these cases the perineal operation succeeds admirably; but it is essential not only to remove the salient obstacle, but to divide its base thoroughly, and drain the bladder for some little time." Such cases are, however, not common; but in the record [2] of two cases of perineal prostatectomy which he has recently performed, one was of this nature, and the result was eminently satisfactory; for immediately after the operation the wall of the bladder, which had lost all power of contraction from over-distension, began slowly to recover.

Dr. G. L. Keyes [3] thinks the perineal route is best where there is great feebleness of the patient, and "where the main indication is to get drainage and to save life," and in which the prostate is not very hard and not very large in its lateral obes.

The operation devised by Dittel, but first performed by Kuster,[4] in which, after a perineal incision, a wedge-shaped piece is excised from the under surface of each lobe of the prostate without opening either the urethra or bladder, has not been very successful. In Kuster's three cases a fistula was left in two, and in the third a vesicula seminalis was opened. The operation is only of use in those rare cases

[1] *Op. cit.* [2] *Lancet*, 1892, vol. ii., p. 142.
[3] *Med. Rec.*, Oct. 31, 1891, p. 525. [4] *Munch. med. Woch.*, Ap. 14, 1891.

where the bladder is very small or rigid, so that the enlarged lateral lobes could not be successfully dealt with by the supra-pubic route.

Perineal prostatectomy, however, although a safer procedure than supra-pubic prostatectomy, is of limited application, and except under the conditions referred to above, the latter method must be adopted. McGill thought that all cases should be treated in this way, inasmuch as it allows of a minute and exact exploration of the vesical surface of the prostate, and of easy and complete removal of the obstructing mass at the same time that it permits of complete and efficient drainage.[1] In only three cases out of twelve upon which McGill operated (by the supra-pubic method) would it have been possible to have removed the obstructing growth through the perineum. Mansell Moullin,[2] in discussing the operation, says "the first question is whether the obstruction will return and render the operation useless; the next whether the bladder can recover." With regard to the first, it is now four years since many of the cases were operated on, and in only one instance has a definite recurrence been noted. The question of recovery of the bladder depends partly upon the condition of the patient as regards general nutrition, partly upon the extent to which the muscular coat has been ruined by over-distension, cystitis, and the repeated use of catheters. The large majority of cases undoubtedly recover the power of expulsion, and in the few failures reported Moullin thinks that "it cannot be too plainly stated that the real reason why the bladder failed was that the operation was performed too late." Sir Henry Thompson [3] says that when habitual catheterism for retention from enlarged prostate has been practised for two years or more, the coats of the bladder lose their power of expelling the contents, and are incapable of regaining it even if the obstruction be removed. The results of supra-pubic prostatectomy have however shown that this is by no means invariable or positive, and it is certainly a strong argument in favour of early operation that the chief palliative measure which replaces it may, within two years, so ruin the bladder that it cannot recover.

Enlargement of the prostate by itself does not require operation in the majority of cases, but in a certain proportion complications set in sooner or later. The catheter has to be passed more and more often, and when passed (sometimes with difficulty) the desire to pass water is as great after the instrument is withdrawn as it was before: cystitis breaks out

[1] TREVES, *Operative Surgery*, vol. ii., 1891, p. 619.
[2] *Brit. Med. Journ., loc. cit.* [3] *Diseases of the Urinary Organs.*

every now and then; attacks of congestion, causing hæmaturia and retention, occur with greater frequency; the urine is becoming offensive; the residuum is becoming larger, and the night's rest is seriously broken. For such as these prostatectomy, by one or other method, according to the special conditions present as indicated above, certainly affords a far better prospect than either cystotomy or drainage, and without sensibly greater risk. On the other hand, if this chance is lost, and the patient allows himself to drift on until his kidneys are diseased, or cystitis has destroyed the muscular power of the bladder, and made it small, hard, and rigid, the conditions are absolutely different. When the capacity of the bladder is much reduced and it cannot be distended, and when under careful treatment it shows no sign of recovery, it is useless attempting to remove the obstruction; cure is impossible, and the risk, if there is the least nephritis, much too great. Palliative measures only can be employed: as much benefit can be derived from drainage as from the most complete enucleation; and no surgeon would undertake a grave operation when one less dangerous to life would answer equally well.

The treatment of tuberculosis of the bladder through a supra-pubic incision has recently[1] been advocated by Dr. James Bell, but he limits the discussion to those severe and advanced cases in which medical and local treatment have failed to give relief, and in which pain and frequent or constant desire to micturate have rendered the patient's life useless, if not burdensome. He considers the advantages of the operation both immediate and remote. The immediate results in all of the cases which he reports gave full relief of the painful and frequent attempts at micturition, the arrest of hemorrhage, and in a short time a cessation of the purulent discharge, except in those cases where pus came from sources other than the bladder ulceration. The remote effects were very much more satisfactory than could have fairly been expected. Of course, when tuberculosis of the kidney already exists, relief of bladder symptoms is all that can be looked for. This operation, moreover, is sound in principle, and is carried out exactly on the same lines as the treatment adopted by surgeons for tubercular disease of other organs; and while it may not give such brilliant results as some joint-operations for example, it would seem, by effecting the removal of the diseased tissue and giving temporary rest

[1] *Journ. Cutan. and Gen.-Urin. Dis.*, Aug., 1892, p. 293.

to the diseased organ, to offer the best, if not the only, hope to many patients who are the subjects of tuberculosis of the bladder. Compared with perineal cystotomy, it gives far more satisfactory rest to the bladder, inasmuch as the urine escapes through a healthy portion of the bladder wall, leaving the inflamed and ulcerated region just within the neck of the bladder free from the irritation of the catheter or drainage tube. This operation, which is neither difficult nor dangerous, and which brings the diseased area directly under the eye and hand, can be relied upon to give more complete and lasting relief to those very distressing conditions produced by ulceration of the mucous membrane of the bladder in the immediate neighbourhood of the outlet than any other method of treatment at present known to surgeons.

In connection with this subject, a preliminary note has just been issued[1] by Dr. Belfield on the use of iodine trichloride in genito-urinary surgery. This substance is made by passing chlorine gas over iodine. The result is a reddish-yellow powder, emitting an odour of chlorine, and readily soluble in water. When dropped into urine, decomposition ensues, and chlorine and iodine are liberated in a nascent state. A similar result occurs with blood, pus and saliva, but the decomposition in these cases takes place more slowly than in urine.

The decomposition appears to be due to mucin, and not to the urinary salts. A 1 per cent. solution rapidly sterilises pure cultures of the staphylococcus pyogenes aureus. Two cases of tuberculosis of the bladder were much improved after injections of 1 per cent. solution; as were also two cases of tuberculous epididymitis treated by hypodermic injections of solutions of $\frac{1}{8}$ to $\frac{1}{4}$ per cent.

The substance has likewise been found useful in checking fermentation in the bladder in cases of residual urine from enlarged prostate.

Dr. Janvrin, in discussing the limitations of vaginal hysterectomy in malignant diseases of the uterus,[2] believes that most operators too readily reject, as unfit for extirpation, all cases in which there has been ever so slight an invasion of the vagina, or in which there seemed to be an extension of malignant disease into the uterine adnexa. He thinks, however, that there are many cases in which the cervix or the body, or both, are diseased, in which the uterus is not

[1] *Journ. Cutan. and Gen.-Urin. Dis.*, Aug., 1892, p. 301.
[2] *Med. Rec.*, July 9th, 1892.

perfectly movable — not from an extension of the carcinomatous condition to the appendages, but simply as a result of old pelvic peritonitis. He is convinced that the appendages do not become involved in a cancerous infiltration until the general condition of the patient is such as to place her beyond any permanent benefit to be derived from the operation. Extension of the disease is much more apt to occur in the *cul-de-sac* by direct infiltration from the cervix, in cases beginning in the cervix, and then into the pelvic peritoneum, than in the adnexa; and also in some cases it will be found even in the cellular tissue between the bladder and uterus.

In giving an opinion as to the proper limitations for this operation, Dr. Janvrin thinks it best to start with some definite idea as to the usual course pursued by cancerous disease of the cervix and uterus, which, he says, is in the following order:

i. Epithelioma developing upon the cervix.
ii. Extension up and into the cervical canal.
iii. Extension up and into the uterine body.
iv. Extension to tissues surrounding cervix.
v. Extension downwards upon vaginal mucous membrane.
vi. Extension downwards upon and through the vaginal wall.
vii. Epithelioma or carcinoma developing primarily upon the endometrium.
viii. Its extension to the body of the uterus.
ix. Its extension to the uterine adnexa.

With this table kept in view, we can safely and justifiably include many cases over and above those in which the cervix, or the cervix and body, are involved; and these additional cases will be confined to instances in which the disease has extended simply to the mucous covering of the vagina (Class V.), and also to those instances in which there is a certain amount of fixation of the uterus by old pelvic inflammatory deposits, but in which the malignant disease is still confined to the uterus itself.

Dr. Janvrin first deals with cases in which the disease has extended downwards *upon* the mucous surface of the vagina. This is generally brought about simply by attrition. A deposit of inflammatory material takes place around it, and separates the malignant deposit from the glandular and cellular structures beyond—the vagina in all respects, excepting as to its mucous membrane, being normal. This condition simply complicates operation by necessitating the removal of the portion of the wall affected, with a half-inch or more of the healthy tissue below. He refers to seven cases of this class in which the disease has not recurred after operation.

He then considers the other class of cases—viz., those in which the disease is located in the body of the uterus, or in the body and cervix, and in which there are also evidences of thickening in the broad ligaments. Here grave doubts as to the propriety of operating may arise, but by the sense of touch, the thickening from simple inflammatory causes always gives a somewhat elastic sensation, while that from cancerous infiltration is always excessively hard and firm. This point in connection with the patient's general condition will usually aid us in selecting cases for operation. This inflammatory thickening, however, renders the operation much more difficult, often absolutely preventing us from drawing the neck of the uterus down to the vulva. He strongly advocates operation, after a careful diagnosis has been made, in either of these two classes.

JAMES SWAIN.

